# The Use of Taurolidine as an Adjunctive Treatment to Reduce Recurrent Peritonitis in Peritoneal Dialysis Patients

**DOI:** 10.7759/cureus.90797

**Published:** 2025-08-23

**Authors:** Gennaro Argentino, Maria Luisa Sirico, Raffaele Genualdo, Lucia Di Micco, Alessandra Antonia Mele, Mario Iorio, Giuseppe Surfaro, Enrica Cascone, Andrea Pota, Andrea Camocardi

**Affiliations:** 1 Department of Nephrology and Dialysis, Ospedale del Mare, Naples, ITA; 2 Department of General Surgery, Ospedale del Mare, Naples, ITA

**Keywords:** biofilm, catheter lock therapy, dialysis-associated infections, multidrug-resistant organisms, peritoneal dialysis, recurrent peritonitis, taurolidine, urokinase

## Abstract

Background and objective

Peritoneal dialysis (PD) represents a cornerstone in the management of end-stage kidney disease (ESKD), offering patients greater autonomy, better preservation of residual renal function, and improved cardiovascular outcomes compared to hemodialysis. However, the technique’s long-term viability is frequently threatened by peritonitis - a complication associated with substantial morbidity, technique failure, and transition to hemodialysis. Among these, recurrent peritonitis is defined as an episode caused by the same organism within four weeks after completion of antibiotic therapy. Taurolidine, a taurine-derived molecule with broad-spectrum bactericidal, fungicidal, and anti-biofilm activity, has demonstrated efficacy in preventing central venous catheter-related infections in hemodialysis patients. Its use in PD remains limited but potentially promising, particularly in the context of biofilm-associated peritonitis. This study aimed to evaluate the safety, feasibility, and clinical efficacy of taurolidine-urokinase lock therapy (TauroLock™-U25,000) in PD patients experiencing recurrent or culture-negative peritonitis, especially those refractory to conventional antibiotic regimens.

Methods

We retrospectively analyzed 13 patients treated at our nephrology centre between 2022 and 2024. Eligible cases included those with recurrent or persistent peritonitis with multidrug-resistant (MDR) organisms or negative cultures suggestive of intraluminal biofilm. Patients underwent three instillations of TauroLock™-U25,000 over 21 days, in combination with standard intraperitoneal antibiotics. Outcomes included clinical resolution, microbiological eradication, recurrence, and adverse events.

Results

All patients completed the protocol without requiring catheter removal. Pathogens included Pseudomonas sp., Staphylococcus aureus, Escherichia coli, Klebsiella sp., Stenotrophomonas maltophilia, and others commonly associated with poor prognoses in PD peritonitis. Two cases were culture-negative. Clinical and microbiological resolution was achieved in 100% of patients, with no relapses during follow-up. Mild, transient abdominal discomfort occurred in eight patients but resolved spontaneously.

Conclusions

Taurolidine-urokinase lock therapy appears to be a viable and safe therapeutic option in the management of peritonitis in PD patients, particularly in those with biofilm-associated or MDR infections. Its favorable tolerability, previously demonstrated in the hemodialysis setting, and the growing evidence of efficacy in PD suggest a potential paradigm shift in catheter-sparing strategies. Further prospective trials are essential to confirm these results and standardize treatment protocols.

## Introduction

Peritoneal dialysis (PD) represents a vital home-based modality for renal replacement therapy, offering patients with end-stage kidney disease (ESKD) important benefits, including preserved residual renal function, greater autonomy, and improved quality of life [1]. PD-associated peritonitis remains a major barrier to the long-term viability of this therapy. The sustainability of PD is frequently jeopardized by this complication, which carries substantial morbidity and mortality risks, contributes to technique failure, increases hospitalization rates, and elevates healthcare costs [1]. The most frequently implicated pathogens are Gram-positive cocci from the skin flora, although Gram-negative organisms are also common. Among these, coagulase-negative staphylococci remain predominant [1]. However, Gram-negative organisms and even culture-negative episodes are increasingly recognized, complicating both diagnosis and treatment [1]. The PD catheter itself constitutes the primary portal of entry, facilitating the development of biofilm-based infections within the peritoneal cavity, a site with limited innate immune defenses [1-2]. Moreover, the warm, glucose-rich dialysate provides an optimal medium for microbial proliferation [1-2].

While most cases of peritonitis respond to intraperitoneal antibiotics, persistent or recurrent episodes, particularly those caused by organisms such as Pseudomonas aeruginosa, often necessitate catheter removal and transition to hemodialysis [1-3]. These infections typically result from biofilms that shield bacteria from antimicrobial agents, making eradication extremely difficult [4]. The formation of intraluminal biofilms represents a paradigm shift in the understanding of recurrent peritonitis [1-3]. These structured microbial communities, embedded in a protective exopolysaccharide matrix, impede antimicrobial penetration and resist immune clearance [1-4]. Consequently, even when initial antibiotic therapy induces temporary remission, residual bacteria within the biofilm may reignite infection, culminating in recurrent peritonitis, defined as infection by the same organism within four weeks of completing therapy [1].

The conventional response to recurrent peritonitis has been catheter removal and temporary or permanent transition to hemodialysis [1-5]. Although effective in interrupting the infectious cycle, this approach undermines the continuity of PD and imposes a psychological and physiological burden on patients [1]. Hence, there is an urgent need for catheter-sparing strategies, particularly those targeting biofilm-associated infections [5].

Taurolidine, a taurine derivative with broad-spectrum bactericidal, fungicidal, and anti-biofilm activity, has demonstrated efficacy as a lock solution in central venous catheters for hemodialysis and parenteral nutrition [6]. Its mechanisms of action include denaturation of microbial cell walls, inhibition of adhesion, neutralization of endotoxins, and disruption of established biofilms [6]. Its use in PD remains limited, although preclinical and clinical data suggest a potential role in managing biofilm-associated recurrent peritonitis [7-8]. When combined with urokinase, a fibrinolytic agent capable of degrading extracellular polymeric substances, the formulation (TauroLock™-U25,000) presents a promising therapeutic strategy for biofilm-mediated peritonitis [8]. Despite encouraging in vitro data and small case series, the use of taurolidine in PD remains largely underexplored [8-9].

This study presents the clinical experience at a tertiary nephrology center utilizing taurolidine-urokinase lock therapy as an adjunct to antibiotics in 13 PD patients with multidrug-resistant (MDR) organisms or culture-negative peritonitis. We aimed to assess the feasibility, safety, and clinical efficacy of this approach.

## Materials and methods

This retrospective, single-center case series was conducted at the Nephrology and Dialysis Unit of “Ospedale del Mare” (ASL Napoli 1 Centro, Naples, Italy), a tertiary referral center with a long-standing PD program. The study was approved by the local ethics committee, and all patients provided written informed consent for treatment and data collection. The study spanned from January 2022 to December 2024 and involved patients (seven males and six females) with a confirmed diagnosis of peritonitis, as defined by the International Society for Peritoneal Dialysis (ISPD) guidelines, caused by either MDR organisms or culture-negative peritonitis highly suggestive of biofilm-associated infection. The inclusion criteria were (1) age >18 years, (2) current participation in the PD program, (3) documented peritonitis episode, and (4) availability of complete clinical, microbiological, and biochemical data before and after treatment. The exclusion criteria were (1) catheter exit-site infection, (2) peritonitis with severe clinical deterioration requiring urgent catheter removal, and (3) inability to tolerate intraluminal therapy.

At baseline, all patients underwent a comprehensive assessment, including physical examination, dialysate effluent analysis, and laboratory evaluation (C-reactive protein (CRP), serum albumin, procalcitonin, and others). The peritoneal equilibration test (PET) was used to classify peritoneal membrane transport status. Dialysis adequacy was reviewed by the PD nursing team in each case. The intervention consisted of three intraluminal instillations of TauroLock™-U25,000 (taurolidine 1.35%, citrate 4%, and 25,000 IU/mL urokinase) over 21 days, corresponding to the average duration of systemic and intraperitoneal antibiotic therapy. Lock volumes were calculated according to catheter lumen capacity (typically 2.0-2.5 mL), and each instillation was retained for at least three hours. To minimize discomfort, the solution was infused very slowly via the PD catheter, following complete drainage of the dialysate. No exit-site infections occurred during the study period.

All patients concurrently received targeted intraperitoneal antibiotic regimens based on antimicrobial susceptibility testing or empirical therapy in culture-negative cases. Antibiotic regimens included intraperitoneal vancomycin, ceftazidime, or meropenem depending on the clinical isolate. In culture-negative cases, vancomycin and ceftazidime were co-administered for a 21-day course, adjusted for residual renal function and dialytic clearance. Clinical outcomes included: (a) resolution of peritonitis signs and symptoms, (b) normalization of inflammatory and nutritional markers (CRP, procalcitonin, and albumin), (c) microbiological eradication, (d) absence of relapse during six months of follow-up, and (e) adverse events related to TauroLock therapy. Data were collected from medical records and follow-up visits.

Statistical analysis

Continuous variables are presented as mean ± standard deviation (SD), and categorical variables as absolute number (N) and percentage (%). Comparisons of pre- and post-treatment laboratory values were performed using the paired Student’s t-test. A p-value <0.05 was considered statistically significant. Test statistics (t values) are provided in the tables where applicable. All analyses were conducted using GraphPad Prism 10.0.

## Results

All 13 patients completed the protocol without the need for catheter removal. Causative organisms included Pseudomonas sp., Staphylococcus aureus, Escherichia coli, Klebsiella sp., Corynebacterium amycolatum (MDR), Stenotrophomonas maltophilia, and others. In two cases, cultures remained negative. Baseline parameters and clinical and biochemical improvements following therapy are detailed in Table 1, which compares key inflammatory markers, albumin, and peritoneal transport categories before and after treatment. The full microbiological spectrum, number of cases per organism, treatment duration, and clinical outcomes are summarized in Table 2.

**Table 1:** Baseline and post-treatment clinical parameters in 13 peritoneal dialysis patients With peritonitis All patients were diagnosed with peritonitis at baseline before starting antibiotic therapy. Data are expressed as mean ± SD. Statistical comparisons were performed using a paired Student’s t-test. Significance was set at p<0.05. The respective t-values and p-values are shown in the table. At baseline, all patients showed elevated inflammatory markers and hypoalbuminemia. After 3 weeks of standardized antibiotic therapy, all patients achieved clinical and biochemical remission. No catheter removals or hospitalizations were reported during the observation period CRP: C-reactive protein; PET: peritoneal equilibration test (categories reflect solute transport rates across the peritoneal membrane): high and high average: fast transporters; average: moderate; low average: slow transporters

Patient	Sex	Age, years	PET category	CRP (baseline)	CRP (post)	t (CRP)	p (CRP)	Albumin (baseline)	Albumin (post)	t (Alb)	p (Alb)
1	M	62	Average	115	6	26.15	0.0	2.8	3.9	-16.39	0.0
2	F	57	High	98	5	26.15	0.0	2.9	3.8	-16.39	0.0
3	M	65	Average	134	4	26.15	0.0	2.5	4.0	-16.39	0.0
4	F	60	High average	102	6	26.15	0.0	2.7	3.7	-16.39	0.0
5	M	59	Average	120	3	26.15	0.0	2.6	3.9	-16.39	0.0
6	F	63	High	110	4	26.15	0.0	2.7	3.8	-16.39	0.0
7	M	61	Low average	140	5	26.15	0.0	2.4	4.1	-16.39	0.0
8	F	58	High average	105	5	26.15	0.0	2.8	3.9	-16.39	0.0
9	M	64	Average	127	3	26.15	0.0	2.6	4.0	-16.39	0.0
10	F	56	Low average	99	4	26.15	0.0	2.9	3.8	-16.39	0.0
11	M	67	Average	138	6	26.15	0.0	2.5	4.2	-16.39	0.0
12	F	59	High average	101	5	26.15	0.0	2.8	3.9	-16.39	0.0
13	M	60	Low average	125	4	26.15	0.0	2.7	4.0	-16.39	0.0

**Table 2:** Microbiological and clinical characteristics of treated cases No inferential statistical analysis was performed due to uniform outcomes. Descriptive statistics were used MDR: multidrug-resistant

Pathogen isolated	N (%)	Clinical outcome	Duration, days	Notes
Pseudomonas sp.	2 (15.4%)	Resolution	21	Descriptive statistics only
Staphylococcus aureus	2 (15.4%)	Resolution	21	Descriptive statistics only
Escherichia coli	1 (7.7%)	Resolution	21	Descriptive statistics only
Klebsiella sp.	1 (7.7%)	Resolution	21	Descriptive statistics only
Kocuria kristinae	1 (7.7%)	Resolution	21	Descriptive statistics only
Corynebacterium amycolatum (MDR)	1 (7.7%)	Resolution	21	Descriptive statistics only
Citrobacter freundii	1 (7.7%)	Resolution	21	Descriptive statistics only
Streptococcus uberis	1 (7.7%)	Resolution	21	Descriptive statistics only
Stenotrophomonas maltophilia	1 (7.7%)	Resolution	21	Descriptive statistics only
Unidentified organism	2 (15.4%)	Resolution	21	Descriptive statistics only

All patients achieved clinical and microbiological resolution (Table 2). All patients completed therapy without requiring catheter removal. No relapses were observed during a median six-month follow-up. Adverse events were minimal; eight patients experienced mild abdominal discomfort that resolved spontaneously. Clinical and microbiological outcomes were evaluated, including pathogen eradication, symptom resolution, recurrence, and adverse effects. Adverse events were limited to mild, transient abdominal discomfort in eight patients, resolving spontaneously. No systemic toxicity or hepatic alterations were observed.

## Discussion

The results support the hypothesis that taurolidine-urokinase lock therapy is a viable adjunctive treatment in PD peritonitis. Peritonitis remains the Achilles’ heel of PD, representing a primary cause of technique failure [1-3]. Persistent infections often result from bacterial biofilms, which significantly hinder antibiotic penetration and treatment efficacy [4-5]. This has made catheter removal the standard response in cases of recurrent peritonitis [5]. By disrupting biofilm integrity and augmenting antibiotic access, taurolidine may facilitate bacterial eradication without catheter removal [6]. Taurolidine’s established use in hemodialysis for catheter-related infections [7] and its anti-biofilm properties [5] support its potential utility in PD.

These findings align with previous reports demonstrating efficacy in S. epidermidis infections [8] and in vitro data against Pseudomonas biofilms [9]. Although taurolidine raised initial safety concerns, more recent studies have shown its acceptable toxicity profile [10-11]. While Demoulin and Goffin explored urokinase alone with partial success [12], our study reinforces the hypothesis that a combination of taurolidine and urokinase may enhance efficacy through synergistic disruption of biofilm structure and thrombotic debris. To our knowledge, the literature on the combined use of taurolidine and urokinase remains scarce. One study has described the use of taurolidine (TauroLock™ HEP 500) in five patients with recurrent peritonitis caused by Gram-positive bacteria, reporting favorable outcomes; however, in the case caused by Staphylococcus aureus, the treatment was ineffective and catheter removal was required [13]. Another study reported the successful elimination of an aggressive Gram-negative organism such as Pseudomonas following taurolidine-urokinase PD catheter lock therapy, with no relevant adverse events [14].

Given the interest in evaluating the biocompatibility of taurolidine and its potential efficacy as an antibacterial agent, Akkuş et al. investigated the effects of intraperitoneal taurolidine lavage on primary colonic anastomosis in a rat model of secondary peritonitis [10]. Peritonitis was induced in 40 rats using an intraperitoneal injection of Escherichia coli. Following colonic resection and anastomosis, rats received a lavage with either taurolidine or saline. On postoperative days three and seven, bursting pressure and tissue hydroxyproline levels were significantly higher in the taurolidine group (p<0.05), indicating enhanced anastomotic strength. Histological analysis also revealed increased fibroblast activity [10]. These findings suggest that taurolidine may improve anastomotic healing and reduce surgical risks in the context of secondary peritonitis [10]. In our series of 13 patients, we successfully treated Gram-positive, Gram-negative, culture-negative, and emerging bacterial peritonitis, and no catheter required removal.

While urokinase alone has shown partial success in previous studies, its combination with taurolidine appears to provide synergistic effects and may be the key to successful catheter rescue. In our series, the intervention was generally well tolerated, as confirmed by the clinical outcomes. All patients completed the protocol without requiring catheter removal, and no relapses were observed during a median follow-up of six months. Adverse events were limited to mild abdominal discomfort in eight patients, which resolved spontaneously without treatment, and no systemic toxicity or hepatic alterations were reported. These observations support the acceptable safety profile of taurolidine-urokinase lock therapy in the clinical setting. Importantly, our findings extend beyond preclinical evidence, demonstrating that the intervention can be safely administered to PD patients with recurrent or biofilm-associated peritonitis. Although the sample size was limited, the consistency of results across a heterogeneous microbial spectrum reinforces the feasibility and tolerability of this approach in real-world practice.

The limitations of the study include the retrospective design, small sample size, and absence of a control group. Nonetheless, the consistency of outcomes across a heterogeneous microbial spectrum underscores the potential role of taurolidine-urokinase therapy in routine nephrological practice.

## Conclusions

Our findings suggest that taurolidine-urokinase lock therapy is a safe and potentially effective adjunct in PD peritonitis, especially in biofilm-associated and MDR infections. It may allow catheter salvage and prevent unnecessary transition to hemodialysis. Taurolidine-urokinase lock therapy demonstrated safety, feasibility, and apparent efficacy in salvaging PD catheters in peritonitis, particularly in cases caused by difficult-to-treat or biofilm-forming organisms. Its use may obviate the need for catheter removal, thereby preserving technique longevity and improving patient quality of life. Prospective trials are needed to validate these findings and optimize treatment protocols.
